# Medical Intelligence

**Published:** 1827-01

**Authors:** 


### INTELLIGENCE, &c.

### CIRCULATION AND ABSORPTION.

Dr. Barry's writings and lectures have set a number of experimenters a work, and the medical societies of the metropolis have become the theatres of the most animated and interesting discussions on the influence of atmospheric pressure in the circulation of the blood and the absorption of fluids. All the experiments hitherto made have confirmed the fact that, at each inspiration, an attempt at a vacuum is made in the chest, though the amount of that attempt—or, in other words, of atmospheric pressure brought into play, was variously estimated by different experimenters. Mr. Ellerby contended, that the influence of this agent must be exceedingly small, since it was incapable of counteracting the power of gravitation, when the animal was in the erect posture. In the horizontal posture of the animals, the fluid was sucked up rapidly through a tube inserted in the jugular vein. Dr. Barry very properly admitted, that atmospheric pressure was only one of the agents in the circulation of the blood, and that the *vis a tergo* was the primary and principal force. In the various discussions Dr. B. had a difficult task to perform, having to contend, as an individual, with scores of antagonists. It was abundantly evident, however, that Dr. B. was not merely *instar omnium*, but that he had infinitely the superiority in argument, extent of research, philosophical accuracy, knowledge of the laws of physics —and last, not least, oratorical eloquence. In this last respect particularly, Dr. B. exhibited a remarkable contrast with not a few of those who ventured to break a lance with him in the contest. In these discussions, however, the dignity of philosophy was maintained, and the very best feeling prevailed on all sides. We have no doubt that the question will be set at rest in the course of the present investigation.

We are gratified to learn, that this distinguished physician has taken up his residence in this metropolis, where he intends to give lectures on the above interesting subjects. We recommend all students to attend these lectures. They will feel the benefits of them the longest day they live.

### Mr. LIZARS' WORK.

Several subscribers have written to us to enquire whether Mr. Lizars means to publish any of the physiological and pathological observations promised in a note to the 10th part. Mr. L. will doubtless answer these queries in our next number.

Dr. Spurzheim is about to commence a Course of Lectures in this metropolis, after lecturing to a large class at one of our principal universities. The loud crowings and clappings of the *Little Cock of the North* have had no effect on the course of phrenology.

### LITERARY NOTICES.

*Ornithology.*—A prospectus has been published of a highly important work on this branch of natural history, by Sir William Jardine, Bart. and P. J. Selby, Esq. the author of the splendid work on British Ornithology, who are to have the co-operation of the most distinguished naturalists in the country. Their plan is to give coloured plates of all the known or most remarkable birds, accompanied by descriptions; and those who are engaged in this undertaking, having access to all the richest, and most splendid collections in the country, are sanguine enough to hope, that the present work will contain the most complete and accurate body of natural science that has ever been published on this subject, and that the plates, by their beauty, finish, and truth of colouring, will be found to illustrate the descriptive part of the work, and to delight the taste, as well as to inform the understanding. The drawings and engravings will be made by the authors, and the plates will be carefully coloured, and finished from living specimens, wherever they can be obtained. Particular attention will be given to the general distinctions and peculiarities of structure, and it is hoped, from the unusual opportunities of information, which the contributors to this work unquestionably possess, that it will be considered a national undertaking. The work will be published in quarterly parts, and the first part will appear early in January, 1827.

---

To be published on the 1st of February, with numerous engravings on wood, Dr. Arnott's work on General and Medical Physics.

It is a system of Natural and Experimental Philosophy, with strictly scientific arrangement, but made easily intelligible to those who have never learned, or who have forgotten the mathematics. In addition to a great mass of illustrations from general nature and the arts, adapted to the present more comprehensive scale of a liberal education, it comprises many very interesting particulars furnished by examination of the animal body under health, disease, and medical treatment; and among these there are new disquisitions and suggestions.

---

In the press, a new edition of the Meteorological Essays, by James Frederick Daniell, Esq. F. R. S.

This edition, besides the former Essays, upon, I. The Constitution of the Atmosphere;—II. The Construction and Uses of a new Hygrometer;—III. The Radiation of Heat in the Atmosphere;—IV. The Horary Oscillation of the Barometer;—V. The Climate of London, with corrections and additions, will comprise Essays on the following subjects:—VI. Evaporation as connected with Atmospheric Phenomena;—VII. Artificial Climate considered with regard to Horticulture;—VIII. The Connexion between the Oscillations of the Barometer at distant places;—IX The Insinuation of Air into the Toriccillian Vacuum, and the Means of preventing the gradual Deterioration of Barometers; it will also contain various Meteorological Observations and Remarks, and numerous Tables, Plates, and Diagrams.

---

### TROPICAL CLIMATES.

The Influence of Tropical Climates on European Constitutions; to which is now added, an Essay on Morbid Sensibility of the Stomach and Bowels, as the proximate Cause or characteristic Condition of Indigestion, Nervous Irritability, Mental Despondency, Hypochondriasis, &c. &c.—preceded by Observations on the Diseases and Regimen of Invalids on their Return from Hot and Unhealthy Climates. By JAMES JOHNSON, M. D. of the Royal College of Physicians, and Physician to His Royal Highness the Duke of Clarence. Fourth Ed. greatly enlarged and improved, 8vo, pp. 680, 1827.

N. B. The Essay on MORBID SENSIBILITY OF THE STOMACH AND BOWELS, &c. is published separately, price 5s. for the benefit of those who may be in possession of the former Editions, or who may not desire to possess the work on Tropical Climates.

### HOSPITAL REPORTS.

Our readers are aware that, in almost all the public hospitals of Paris, some distinguished ELEVE INTERNE is deputed by each physician or surgeon, to draw up a quarterly report of the diseases, to be published in some of the various medical journals of the French metropolis. This procedure is productive of many advantages. In the first place, the public at large are incalculably benefited—in the second place, the medical officers of the hospitals are fairly represented, and their practice made widely known—in the third place, the pupil, who thus reports, is stimulated to acute and careful observation of the phenomena of disease, his mind is strengthened by exercise, his accuracy is guaranteed by the responsibility attached to the undertaking, and he is thus early introduced to public notice, and favourably so, if his reports are ably drawn up. We have, on many occasions, held up this example to the medical institntions of this country, metropolitan and provincial; but it has not yet been acted on, in the manner we have described. Wishing to encourage such an undertaking, by giving it a commencement, we hereby offer to devote about a sheet (16 to 24 pages) of closely printed letter press, in the EXTRA-LIMITES of each number, to the BEST quarterly report which may be transmitted to us, from a metropolitan or large provincial hospital, adjudging to the reporter a complete set of this Journal, and a continuation of the Journal, free of expense, for two years, with a perpetual registry of his name in a permanent list set apart for that purpose, and kept standing in a conspicuous part of the Journal. The prize is small, in pecuniary value; but the testimony of merit will be thus placed in a more conspicuous point of view than has ever yet been done.

